# Non-canonical targets destabilize microRNAs in human Argonautes

**DOI:** 10.1093/nar/gkx029

**Published:** 2017-01-23

**Authors:** June Hyun Park, Sang-Yoon Shin, Chanseok Shin

**Affiliations:** 1 Department of Agricultural Biotechnology, Seoul National University, Seoul, 08826, Republic of Korea; 2 Interdisciplinary Program in Agricultural Genomics, Seoul National University, Seoul, 08826, Republic of Korea; 3 Research Institute of Agriculture and Life Sciences, Seoul National University, Seoul, 08826, Republic of Korea; 4 Plant Genomics and Breeding Institute, Seoul National University, Seoul, 08826, Republic of Korea

## Abstract

Although much is known about microRNA (miRNA) biogenesis and the mechanism by which miRNAs regulate their targets, little is known about the regulation of miRNA stability. Mature miRNAs are stabilized by binding to Argonaute (Ago) proteins, the core components of the RNA-induced silencing complex (RISC). Recent studies suggest that interactions between miRNAs and their highly complementary target RNAs promote release of miRNAs from Ago proteins, and this in turn can lead to destabilization of miRNAs. However, the physiological triggers of miRNA destabilization with molecular mechanisms remain largely unknown. Here, using an *in vitro* system that consists of a minimal human Ago2–RISC in HEK293T cell lysates, we sought to understand how miRNAs are destabilized by their targets. Strikingly, we showed that miRNA destabilization is dramatically enhanced by an interaction with seedless, non-canonical targets. We then showed that this process entails not only unloading of miRNAs from Ago, but also 3΄ end destabilization of miRNAs occurred within Ago. Furthermore, our mutation analysis indicates that conformational changes in the hinge region of the Ago PAZ domain are likely to be the main driving force of the miRNA destabilization. Our collective results suggest that non-canonical targets may provide a stability control mechanism in the regulation of miRNAs in humans.

## INTRODUCTION

MicroRNAs (miRNAs) are ∼22-nucleotide (nt), small regulatory RNA molecules that play important roles in a wide range of biological processes. miRNAs are transcribed as primary miRNA (pri-miRNA) transcripts that are processed via two cleavage steps that are mediated by Drosha and Dicer (1,2). These tandem actions convert pri-miRNAs into precursor miRNAs (pre-miRNAs) and finally results in the production of the ∼21–23 nt miRNA duplexes. The miRNA duplexes, which contain a 5΄ phosphate and a 2-nt 3΄ overhang on each end, are subsequently loaded into Argonaute (Ago) proteins with the aid of chaperone machinery (3,4). The two strands of the duplex are separated within the Ago proteins. One of the strands is retained as the guide, whereas the other, the passenger strand, is cleaved (5) and/or ejected (6,7). The seed region (nt 2–8) of mature miRNAs directs the RNA-induced silencing complex (RISC) to target mRNAs by binding to complementary sequences (8), which results in mRNA destabilization and/or translational repression (9,10).

Precisely controlled expression of miRNAs is important to ensure that their targets are repressed properly. Although much is known about miRNA biogenesis and its regulation, especially at the level of the pre-miRNAs (11–13), relatively little is known about how functional, mature miRNAs are turned over and degraded. Once loaded into Ago proteins, miRNAs are stabilized (14,15), with half-lives raging from hours to days (16). However, mounting evidence suggests that they are also subjected to active regulation under specific cellular contexts, including development, differentiation, viral infection and in response to stimuli (17–20). These observations raise intriguing questions regarding the nature of the general triggers affecting miRNA stability. Because 5΄ and 3΄ ends of miRNAs are bound to the MID and PAZ domains of Ago, respectively (21,22), they are likely to require dissociation from Ago in order to become susceptible to degradation by nucleases (*i.e*., miRNA destabilization). Recent findings suggest that interactions between miRNAs and their highly complementary targets promote miRNA destabilization and release from Ago proteins (23), which is accompanied by the accumulation of 3΄ miRNA isoforms (24,25), although the detailed mechanisms underlying such target RNA-directed miRNA destabilization remain largely unknown.

Here, using an *in vitro* system that consists of a minimal Ago2–RISC in cell lysates, we sought to understand how miRNAs in human Ago proteins are destabilized by their targets. During the course of our studies, surprisingly, we found that seedless, non-canonical targets, which are increasingly recognized as being more widespread than initially anticipated (26–30), destabilize miRNAs in human Ago proteins. We also demonstrated that the target-directed mechanism entails not only unloading but also 3΄ end destabilization of miRNAs within Ago, which is driven by the dynamic nature of the L1-PAZ domain. Furthermore, we analyzed target sequence constraints in detail, and showed that extensive 3΄ pairing is primarily responsible for conferring the specificity of non-canonical interactions. Our combined results provide novel mechanistic insights into the dynamic interplay between miRNAs and their targets, which increase our understanding of how miRNAs are regulated in humans.

## MATERIALS AND METHODS

### Cell culture

HEK293T and HeLa S3 cells were cultured in Dulbecco's modified Eagle's medium that was supplemented with 10% (v/v) fetal bovine serum (FBS), 100 U/ml penicillin and 100 μg/ml streptomycin at 37°C in an atmosphere with 5% CO_2_. *Drosophila* S2 cells were cultured at 25°C in Schneider's medium supplemented with 10% FBS.

### Cell lysate preparation

HEK293T cells at 30–50% confluence were transfected with FLAG-tagged Ago expression plasmids (10 μg per 100-mm dish) using the calcium phosphate method, and they were harvested after 48 h. Cytoplasmic lysates from HEK293T cells were prepared essentially as described (6). To obtain the expression plasmids encoding the FLAG-tagged human Ago proteins, the coding region of each cDNA fragment was inserted into pcDNA-based vectors (Invitrogen). The plasmids for hAgo1 and the catalytic (D597A) and hinge (F181A) mutants of hAgo2 were generated previously (6). The PAZ (H271A, R277A, K278A, R280A, Y311A, H316A, Y311A/H316A, H271A/Y311A/H316A, R277A/K278A/R280A, Δ277-280) and L1-PAZ hinge (F181A/Y311A) mutants of hAgo2 were generated by site-directed mutagenesis using the primers listed in Supplementary Table S1.

### *In vitro* target RNA-directed miRNA destabilization assay

miRNAs were assembled into Ago2–RISC under standard *in vitro* RNAi conditions (31) that typically contained 2.5 μl of cell lysate, 1.5 μl of reaction mix (31) and 0.5 μl of 100–200 nM of radiolabeled miRNA duplex (5΄-^32^P-radiolabeled guide strand annealed to an unlabeled phosphorylated passenger strand) at 37°C for 1 h. The final concentration of Mg^2+^ was 1.5 mM. After miRNA assembly, an excess synthetic target RNA or 5΄ capped and poly (A) tailed mRNA (10–100 pmol, saturating at ≥1 μM) was added and further incubate at 37°C for 1–2 h. The reactions were stopped by adding an equal volume of 2× formamide dye containing 25 mM ethylenediaminetetraacetic acid (EDTA) and 0.1% sodium dodecyl sulphate (SDS), followed by heating at 95°C, and they were resolved by electrophoresis through a 15% urea/polyacrylamide sequencing gel, under highly stringent denaturing conditions (32). For the native gel analysis in Figure 7C, Ficoll was added to 3% (f.c.) after the reactions were terminated, followed by resolution by 15% native polyacrylamide gel electrophoresis (PAGE) containing 1.5 mM Mg^2+^ in both the gel and running buffer. Phosphorimaging was performed using a BAS-2500 image analyzer (Fujifilm, Tokyo, Japan), and the signal intensities of the full-length miRNAs were quantified for the relative 3΄ end stability using MultiGauge (Fujifilm). The target RNA sequences are listed in Supplementary Table S1.

### Target-directed miRNA destabilization in cells

Six-well plates were seeded with 4–6 × 10^5^ cells 16–24 h prior to transfection. HEK293T cells were initially transfected with 10 nM of miRNA duplex, 1 μg of FLAG-Ago2 plasmid (pcDNA3.1), followed by transfection with 100 nM of target RNA or 1 μg of 4× target expressing plasmid (pIS2, Addgene plasmid #12177) at 6 and/or 24 h after the initial transfection. For endogenous miRNA targeting, cells were transfected with 100 nM of target RNA. Lipofectamine 2000 was used for transfections according to the manufacturer's protocol (Invitrogen, Carlsbad, CA, USA). At 48 h after the initial transfection, cells were washed three times with cold phosphate buffered saline and lysed in buffer containing 20 mM HEPES-KOH, pH 7.4, 150 mM KOAc, 1.5 mM Mg(OAc)_2_, 0.1% Triton X-100, 5% glycerol and 1× EDTA-free protease Inhibitor Cocktail (Roche, Basel, Switzerland). Cell lysates were incubated for 10 min on ice and cleared by centrifugation at 15,000 rpm for 20 min at 4°C. Total cytoplasmic RNA was extracted using TRI reagent (Ambion, Thermo Fisher Scientific, Waltham, MA, USA). For Ago2 immunoprecipitation, cell lysates were incubated with anti-FLAG M2 affinity gel (Sigma-Aldrich, St. Louis, MO, USA) for 12–16 h with gentle rocking at 4°C, followed by four washes with 10× bead-volumes of IP wash buffer containing 20 mM HEPES-KOH, pH 7.4, 300 mM KOAc, 1.5 mM Mg(OAc)_2_, 0.01% Triton X-100 and 1× EDTA-free protease Inhibitor Cocktail. RNA was recovered from the beads using TRI reagent for subsequent detection by northern hybridization.

### Northern hybridization

RNAs were resolved in a 12.5% denaturing polyacrylamide gel, electrophoretically transferred to Hybond-N+ membrane (GE Healthcare, Little Chalfont, UK) and UV cross-linked at 0.12 J/cm^2^. The DNA probe (Supplementary Table S1) was radiolabeled using T4 polynucleotide kinase (Takara, Shiga, Japan) and [γ-^32^P] ATP (6,000 Ci/mmole, PerkinElmer), and it was hybridized with the membrane using PerfectHyb Plus (Sigma-Aldrich) at 37°C overnight. The hybridize membrane was washed with Buffer I (2× saline-sodium citrate (SSC) and 0.1% SDS) and Buffer II (0.5× SSC and 0.1% SDS) at 37°C for 25 min each.

### Unloading assay

To prepare immunopurified hAgo2, 50 μl of cytoplasmic lysates from HEK293T cells expressing FLAG-hAgo2 was incubated with 20 μl of anti-FLAG M2 Affinity Gel (Sigma-Aldrich) for 2–4 h with gentle rocking at 4°C, followed by six washes with 10× bead-volumes of wash buffer containing 20 mM HEPES-KOH, pH 7.4, 150 mM KOAc, 1.5 mM Mg(OAc)_2_, 0.1% Tween-20 and 1× EDTA-free protease Inhibitor Cocktail. The immunopurified hAgo2 was loaded with 20 nM of a single-stranded, 5΄-^32^P-radiolabeled miRNA at 37°C for 1 h, followed by six washes to remove unbound RNAs. Immobilized hAgo2 was eluted from the resin with 300 μg/ml of 3× FLAG peptide (Sigma-Aldrich) for 1 h with gentle rocking at RT. The unloading reactions were performed essentially as described as previously (23). Briefly, miRNA-bound Ago2 was incubated with 5 μM of target RNA at 37°C for 1 h in a 5-μl reaction volume. After the unloading reactions, Ficoll was added to 3% (f.c.), and the samples were directly loaded onto 15% native polyacrylamide gels containing 1.5 mM Mg^2+^ in both the gel and running buffer.

### Ago2 cleavage assay

To prepare targets, DNA fragments containing the target site were amplified by the polymerase chain reaction, *in vitro* transcribed and radiolabeled at the 5΄-cap by guanylyl transferase and [α-^32^P] GTP (3000 Ci/mmole, PerkinElmer, Waltham, MA, USA) using the mScript mRNA production system (Epicentre, Madison, WI, USA) according to the manufacturer's instructions, followed by denaturing polyacrylamide gel purification. A total of 50 nM 5΄-phosphorylated small RNA duplex was pre-incubated prior to the addition of ∼5 nM ^32^P-cap-radiolabeled target RNA. For the cleavage assay in Figure 5C and Supplementary Figure S6A, the radiolabeled miRNAs were first destabilized by cold targets prior to the addition of cap-radiolabeled perfect targets. The reactions were stopped by adding an equal volume of 2× formamide dye containing 25 mM EDTA and 0.1% SDS, and then they were resolved in a 15% denaturing polyacrylamide gel.

### *In vitro* RISC assembly assay

*In vitro* RISC assembly assays were performed essentially as described previously (6,33). A total of 10 nM of guide strand radiolabeled duplexes (*i.e*., a 5΄-^32^P-radiolabeled guide strand annealed to an unlabeled phosphorylated passenger-strand) was incubated in the standard reaction mixture with 10 nM of 2΄-*O*-methyl antisense oligonucleotide (ASO) as a target for native gel analysis (Figure 7B and Supplementary Figure S4C). For an alternative native gel analysis (Figure 4B), the 2΄-*O*-methylated ASO was radiolabeled instead of the guide strand (Supplementary Table S1). The RISC complexes were resolved by vertical, native 1.4% agarose gel electrophoresis at 300 V in a 4°C cold room.

### Antibodies

The primary antibodies included polyclonal rabbit anti-FLAG (1:5,000; Sigma-Aldrich), monoclonal mouse anti-hAgo2 (1:500; Abcam, Cambridge, UK), polyclonal rabbit anti-α-tubulin (1:15,000; Abcam). The secondary antibodies for chemiluminescent detection were horseradish peroxidase-conjugated goat anti-rabbit (or anti-mouse) IgG antibodies (Jackson ImmunoResearch, West Grove, PA, USA).

## RESULTS

### miRNAs are stable in Argonaute but they are destabilized upon non-canonical target binding

**Figure 1.**
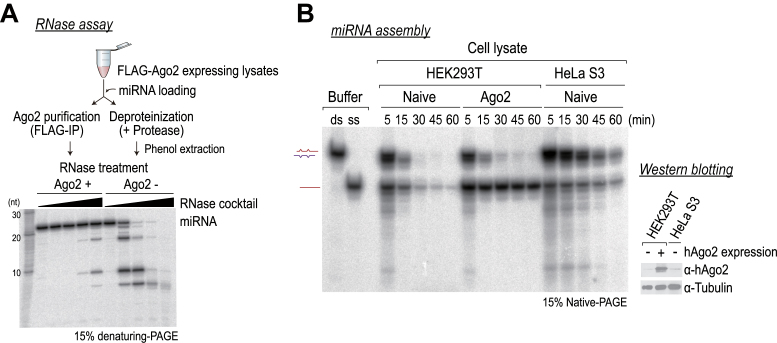
miRNAs are stabilized by Argonaute (Ago). (**A**) Top: schematic of the experiment. Bottom: 5΄-^32^P-radiolabeled miRNAs were subjected to RNase (RNase A and T1) treatment in the presence (Ago2+, immunopurified Ago2) or the absence of Ago (Ago2–, deproteinized), and they were analyzed in 15% denaturing-PAGE. (**B**) Left: miRNA duplexes were assembled in lysates from naïve HEK293T cells, from cells expressing Ago2, or from HeLa S3 cells and they were analyzed by 15% native-PAGE. ds, miRNA duplex; ss, single-stranded miRNA. Right: western blot analysis using an anti-hAgo2 antibody. Anti-tubulin served as an internal control.

It has been widely considered that Ago proteins stabilize mature miRNAs. We hypothesized that if Ago proteins are degraded by proteases in cells, miRNAs should be rapidly degraded by nucleases (*i.e*., miRNA destabilization). To test this idea *in vitro*, we immunopurified Ago2 complexes and subjected them to protease digestion, followed by RNase treatment (Figure 1A). miRNAs were degraded rapidly by RNases in the protease-treated control, whereas those in Ago2 complexes were largely protected from degradation, suggesting that Ago protein is required for mature miRNA stability (Figure 1A).

To explore the mechanism of how targets destabilize miRNAs in human Ago proteins, we used cell lysates prepared from human embryonic kidney 293T (HEK293T) cells that express epitope-tagged human Ago2 (hAgo2). HEK293T cells are often used for exogenous RISC programming because a naïve HEK293T cell lysate on its own is not competent to reconstitute RNA interference (RNAi) *in vitro* (6,34) (Supplementary Figure S1A). We assembled miRNA duplexes in cytoplasmic lysates from naïve HEK293T cells or from those expressing Ago2, and we observed their maturation by native PAGE (Figure 1B). In naïve lysates, single-stranded, mature miRNAs were unstable and presumably degraded by endogenous nucleases, while those of Ago2-expressing lysates remained stable and exhibited greater stability than those of HeLa cell lysates (Figure 1B).

**Figure 2.**
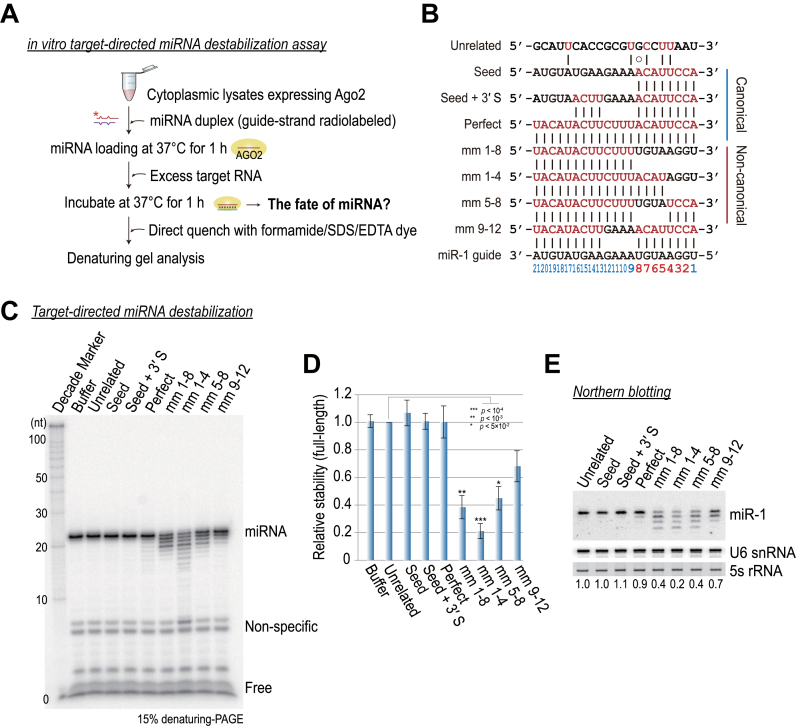
*in vitro* recapitulation of target RNA-directed miRNA destabilization in human Ago2. (**A**) A schematic illustration of the *in vitro* assay for target-directed miRNA destabilization. The asterisk indicates a 5΄-^32^P radiolabel. (**B**) Schematic of paired miRNAs and complement target RNAs. Targets lacking the canonical perfect seed match are considered to be non-canonical. (**C**) Non-canonical targets destabilize miRNAs in Ago2–RISC. (**D**) Quantitation of (C). The means ± standard deviations (SDs) for four independent replicate experiments are shown. The *P*-value was calculated with a Student's *t*-test. (**E**) Northern hybridization, which was performed with samples as described (A), but used cold miRNA duplexes. The blot was probed for U6 snRNA as a loading control. Ethidium bromide-stained 5S rRNA served as another loading control. The numbers below the blot are the relative expression levels, normalized using the U6 snRNA control.

To examine the fate of miRNAs in Ago2–RISC after binding their targets, we added excess synthetic target RNAs after 1 h of exogenous miRNA programming (Figure 2A). These target RNAs included canonical targets (with intact seed matches) and recently reported non-canonical targets (26–30) that usually contain no, or imperfect, seed matches that are compensated by extensive 3΄ pairing (Figure 2B). The reaction was allowed to proceed for an additional 1 h at 37°C, and then it was quenched directly with formamide dye at 95°C (without ethanol precipitation), which enabled us to detect free RNA species, while ensuring equal loads, during denaturing gel electrophoresis. Surprisingly, the results from our newly developed assay showed that miRNAs were severely destabilized by non-canonical targets in a time- and concentration-dependent manner, irrespective of whether the targets were synthetic or *in vitro*-transcribed mRNAs (Figure 2C and Supplementary Figure S1B–D). These results contrast with those for the canonical targets, which did not have significant, adverse effects on miRNA stability (Figure 2C). Excess target RNA addition by itself did not affect miRNA stability (unrelated target control) (Figure 2C). Next, we quantified the level of the full-length miRNAs in multiple replicates, and we concluded that the effects of non-canonical targets were statistically significant (*P* < 10^−3^) and reproducible (Figure 2D). Northern hybridization, which was performed with samples as described in Figure 2A, but which used cold miRNA duplexes, confirmed that the results of our *in vitro* assay quantitatively reflected the levels of miRNAs and that the *in vitro* assay exhibited higher sensitivity (Figure 2C and E). Thereafter, we exploited this experimental system to further biochemically dissect how target RNAs destabilize miRNAs in human Ago2–RISC.

### miRNA seed pairing is important for miRNA stability

**Figure 3.**
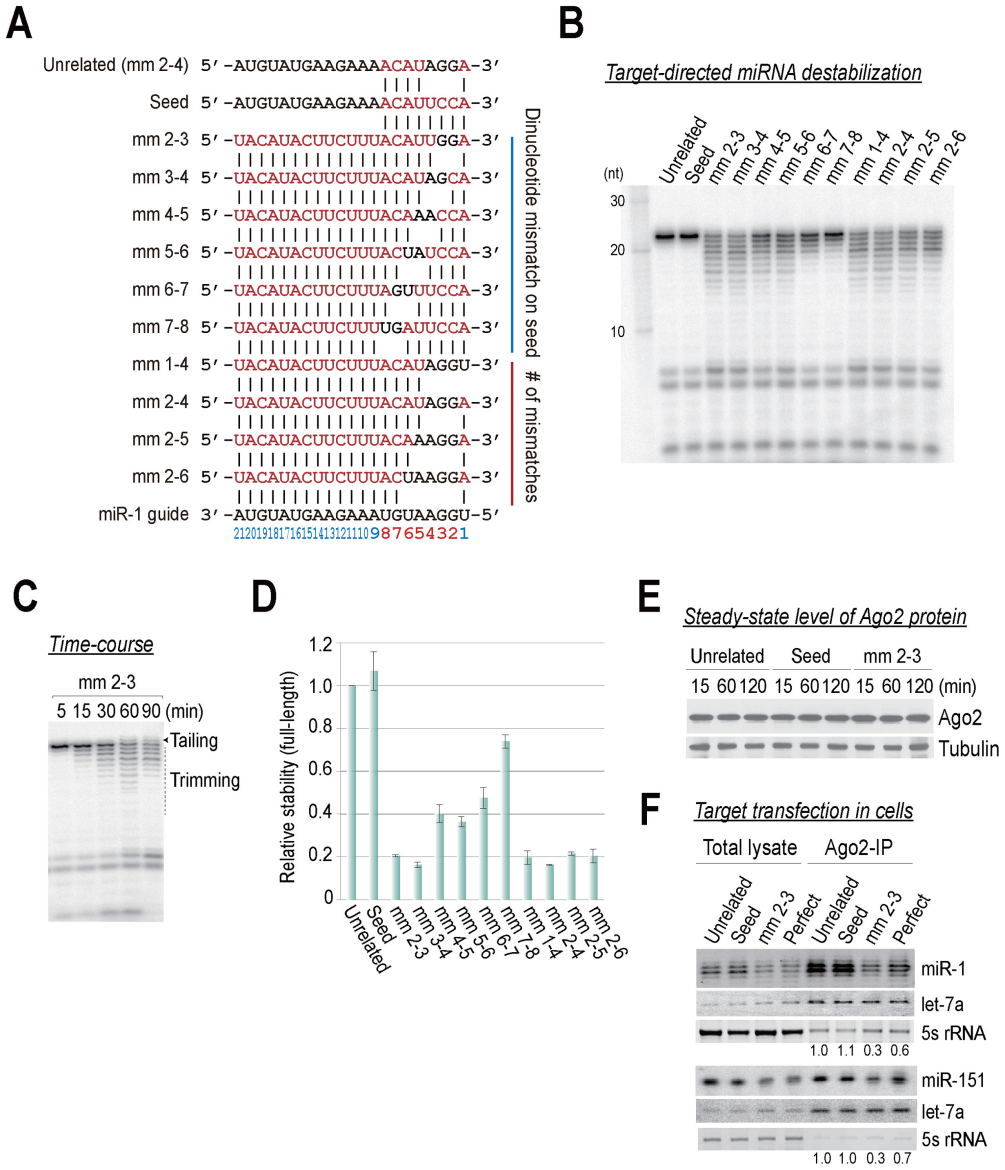
miRNAs are destabilized by seedless, non-canonical targets. (**A**) Schematic of paired miRNAs and complement target RNAs. (**B**) The effects of dinucleotide mismatches in the seed region on miRNA stability. (**C**) Time-course analysis of (B); the mm 2–3 target serves as a representative example. (**D**) Quantitation of (B). The means ± SDs for two independent replicate experiments are shown. (**E**) Western blot analysis using an anti-FLAG antibody confirmed the expression of the tagged Ago2 protein. Anti-tubulin served as an internal control. (**F**) HEK293T cells were transfected with 10 nM miRNA duplex, 100 nM target RNA and FLAG-Ago2 expression plasmid. Cell lysates were subjected to FLAG-IP, followed by northern blotting using the miR-1 or miR-151 probe. The numbers below the blot are the relative expression levels, normalized using let7a as a loading control. Ethidium bromide-stained 5s rRNA served as another loading control.

Our initial results indicated that the seed region is not only important for target silencing, but also for miRNA stability. To more precisely examine the effect of seed matches on the stability of miRNAs, we continued our analysis of target RNAs containing dinucleotide mismatches in the seed region (Figure 3A). A different, unrelated control (seed mismatch without 3΄ pairing) showed no effects on miRNA stability, presumably because this type of target rapidly dissociates from Ago2–RISC (35). In contrast, targets with extensive 3΄ pairing drastically destabilized miRNAs (Figure 3B–D), without affecting steady-state levels of Ago2 protein (Figure 3E). Mismatches in seed nucleotides [guide nt 2–8 (g2–g8)] did not contribute equally to the destabilization, but the loss of interactions involving as little as 2–3 nt within g2–g4 seemed to be the most critical (Figure 3D). These results are reminiscent of findings from recent single-molecule studies that showed g2–g4 are important for the initial probing of target sequences (35,36). In addition, we heterologously expressed miRNAs and their target RNAs in HEK293T cells, and then we performed a northern hybridization analysis at 48 h post-transfection, which demonstrated that the miRNAs were destabilized by non-canonical targets in cells (Figure 3F).

### Non-canonical targets trigger the 3΄ end destabilization of miRNAs

**Figure 4.**
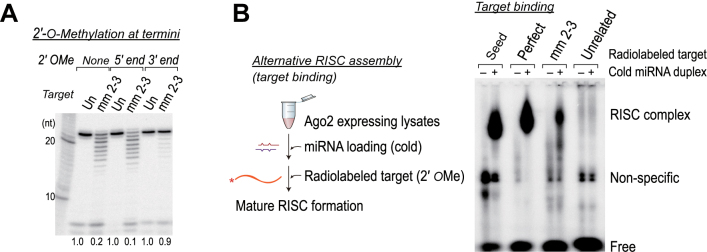
Non-canonical targets trigger the 3΄ end destabilization of miRNAs and bind with less affinity to human Ago2–RISC (**A**) miRNAs are destabilized at their 3΄ ends upon target binding. miRNAs that were 2΄-*O*-methylated either at their 3΄ or 5΄ ends were subjected to the target-directed destabilization assay. Un, unrelated control. (**B**) Left: schematic of the experiment. Right: miRNAs in Ago2–RISC are able to bind non-canonical targets, albeit less efficiently.

To examine whether miRNAs are destabilized from the 5΄-3΄ or 3΄-5΄ direction, we first tried labeling the 3΄ end of the guide strands via ligation with [5΄-^32^P] cytidine-3΄, 5΄-bis-phosphate (pCp). However, miRNA duplexes with a 3΄ pCp on the guide were largely refractory to RISC loading, presumably because the PAZ domain specifically bind to the 3΄ OH ends of miRNA duplexes (data not shown). In an alternative strategy, we used miRNA duplexes whose guide strands were 2΄-*O*-methylated at their 3΄ or 5΄ termini (Figure 4A). We found that 2΄-*O*-methylation of the 3΄ termini of the miRNAs largely protected them from nuclease degradation (Figure 4A), whereas 2΄-*O*-methylation of the 5΄ termini did not. These results suggest that miRNAs are likely to be destabilized at their 3΄ ends upon target binding.

We initially used the miRNA miR-1, which is specially expressed in heart and skeletal muscle, as a model of exogenous miRNA assembly in HEK293T cell lysates. To test whether 3΄ end destabilization is generally applicable to other miRNA sequences, we examined several other miRNA duplexes (Supplementary Figure S2A). The results were broadly consistent across different miRNAs, although each miRNA exhibited a slight different sensitivity (Supplementary Figure S2B). Because the different GC contents of miRNAs affects target recognition, we reasoned that the GC content would also play a role in 3΄ end destabilization, especially for the 3΄ region of miRNAs. To test this idea, we mutated the 3΄ region of miR-1 to increase its GC content to 67%, which is higher than that of most miRNAs (Supplementary Figure S2C). We found no strong correlation between the GC content and the extent of 3΄ end destabilization (Supplementary Figure S2C), although the GC content may have had a small effect.

### Human Ago2–RISC binds seedless, non-canonical targets with extensive 3΄ pairing

Our results raised an intriguing question regarding how non-canonical targets induce miRNA destabilization, because it is commonly believed that miRNAs are less likely to bind targets with an imperfect seed. To investigate whether miRNAs indeed bind to such targets, we performed a RISC assembly assay (33), which was originally intended to exclusively detect the mature RISC. By radiolabeling target RNAs instead of miRNAs, it is possible to monitor RISC complex formation, but this only occurs if the miRNAs are capable of binding targets (Figure 4B). Our analysis indicated that seed pairing alone was usually sufficient for binding, at least for human Ago2 (Figure 4B). In addition, we demonstrated that the miRNAs were able to bind targets with imperfect seed matches and extensive 3΄ pairing, albeit much less efficiently (∼12 ± 5%) than the perfect target control (Figure 4B). Our results may partly explain why non-canonical targets are found in the miRNA interactome (26–30).

### A large fraction of miRNAs is still in Ago2 following target-directed destabilization

Structural studies revealed that miRNAs are tightly bound to Ago proteins and that their 5΄ and 3΄ ends are anchored in MID and PAZ domains, respectively (21,22). Therefore, one can expect that miRNAs should be dislodged from Ago proteins, to make them accessible to exoribonucleases. This idea was first demonstrated in *Caenorhabditis elegans* Ago by Grosshans *et al.* (37), and more directly by a recent study that used recombinant human Ago2 that was immunopurified from *Spodoptera frugiperda* Sf9 cell lysates (23). The results from our *in vitro* assay likely reflect two different possibilities: (i) miRNAs are completely released from Ago and degraded, or (ii) while the 5΄ end of miRNAs are stably anchored in the MID domain, their 3΄ ends are released from the PAZ domain and destabilized (*i.e*., 3΄ end destabilization).

**Figure 5.**
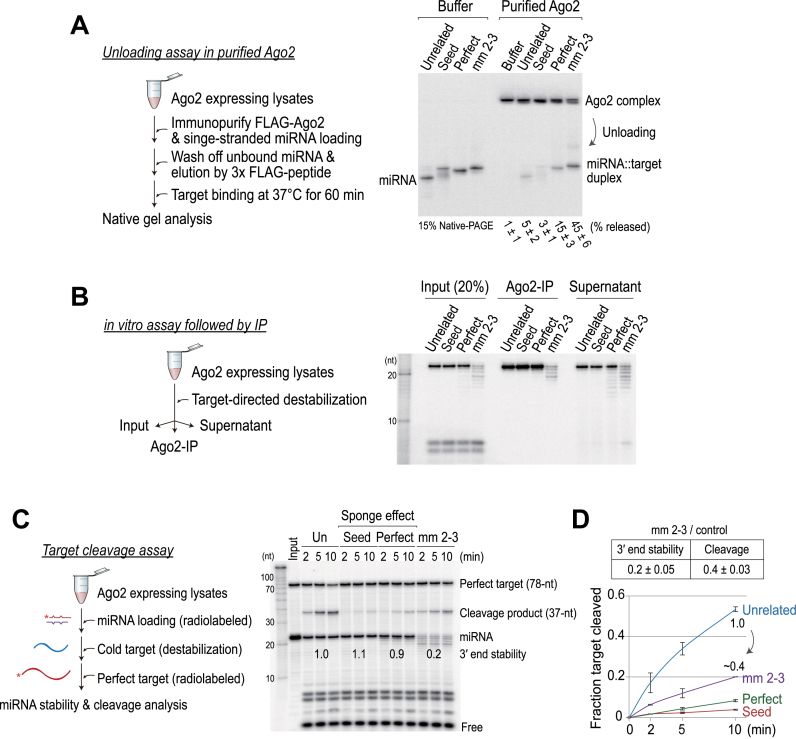
The target-directed mechanism entails a combination of unloading and 3΄ end destabilization in Ago protein. (**A**) Left: schematic of the experiment. Right: large fractions of miRNAs were in Ago2-complexes after non-canonical target binding. In the buffer control, single-stranded miRNAs were annealed to each target RNA at 37°C. (**B**) Left: schematic of the experiment. Right: 3΄ end destabilization occurs in Ago2-bound fraction during non-canonical target binding. (**C**) Left: schematic of the experiment. Right: a substantial fraction of 3΄ trimmed miRNA species in the Ago2-complex can participate in cleavage catalysis. (**D**) Top: miRNAs that are destabilized by non-canonical targets (0.2 ± 0.05) are capable of catalyzing cleavages, whose efficiencies are higher than expected (0.4 ± 0.03). Discrepancies between the two results (3΄ end stability and target cleavage) indicate that many miRNAs are still in Ago2–RISC following target-directed destabilization. Bottom: quantitation of the fraction target cleaved is shown. Data are the mean ± SD for two independent experiments.

To discriminate between these two possibilities, we first examined the contribution of unloading during the target-directed destabilization process. We performed an unloading assay essentially as described (23), except that we used hAgo2 that was immunopurified from HEK293T cell lysates. In addition, we eluted Ago2 proteins from beads and simultaneously analyzed the fractions of miRNAs that are either bound to or released from Ago2 by native PAGE (Figure 5A). Our analysis showed that ∼45 ± 6% of the miRNAs were released from the Ago2 complex by non-canonical targets, whereas most of the miRNAs were stably associated with Ago2 by other targets (Figure 5A). Our *in vitro* assay showed that the 3΄ ends of the miRNAs were destabilized to a greater extent (∼80%) by non-canonical targets (Figure 3D). The discrepancy between these two results indicated that unloading does not fully explain the target-directed destabilization mechanism, and that some parts of the destabilization process occur within the Ago2-complex.

To test this, we performed an *in vitro* assay, after which we fractionated samples via Ago2 immunopurification (Figure 5B). Consistent with the aforementioned idea, we found that 3΄ end destabilizations were observed in Ago2-bound fraction (Figure 5B). This led us to question whether some parts of 3΄ trimmed miRNAs are present in the Ago2-complex, and whether they are functionally active. To answer this question, we performed a target cleavage assay. A conventional cleavage assay includes the assembly of cold miRNA duplexes, followed by the addition of cap-radiolabeled targets. We slightly modified the protocol so that we could monitor the efficiency of target cleavage after 3΄ end destabilization (Figure 5C). Upon canonical target bindings, the cleavage efficiency was minimal, although miRNA stability was not compromised (Figure 5C). These results indicated that most miRNAs in Ago2–RISC were stably associated with cold canonical targets (*i.e*., sponge effects). In contrast, miRNAs that were destabilized by non-canonical targets (0.2 ± 0.05) were capable of catalyzing cleavages, whose efficiencies were higher than expected (0.4 ± 0.03) (Figure 5D). These results support and extend the conclusion that a substantial fraction of 3΄ trimmed miRNAs are in functional Ago2–RISCs that can participate in cleavage catalysis. Based on these collective results, we conclude that at least one-half of miRNAs are still associated with Ago2–RISC following target-directed destabilization.

### 3΄ complementarity confers specificity for target-directed destabilization

**Figure 6.**
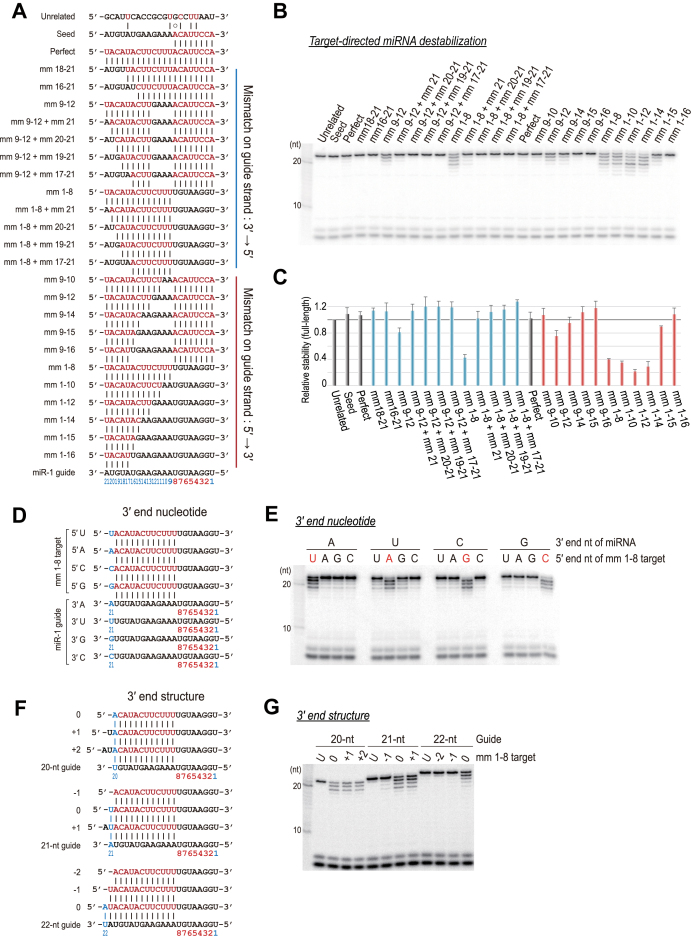
3΄ complementarity confers specificity for target-directed destabilization. (**A**) Schematic of paired miRNA and complement target RNAs. (**B**) 3΄ complementarity confers a specificity of destabilization. (**C**) Quantitation of (B). The mean ± SD for at least two independent experiments is shown. (**D** and **E**) A single mismatch at the 3΄ end nucleotide largely abrogates the destabilization, regardless of the identity of the 3΄ end nucleotide of the guides or (**F** and **G**) the 3΄ end structure of guide–target duplexes.

We showed that 3΄ complementarity is important for miRNA destabilization. To gain additional mechanistic insights into target-directed destabilization, we analyzed target sequence constraints in detail (Figure 6A and Supplementary Figure S3A). From this analysis, we arrived at three main conclusions: (i) a mismatch at the last nucleotide inhibits 3΄ end destabilization (*e.g*., mm 1–8 versus mm 1–8 + mm-21) (Figure 6B and C); (ii) 3΄ end destabilization is mostly diminished when miRNAs are seed matched (*e.g*., mm 9–14 versus mm 2–4 + mm 9–14) (Supplementary Figure S3A); (iii) at least 7 nt or more of 3΄ end complementarity (from mm 1–8 to mm 1–16) are required for destabilization, which occurs largely independently of the slicer activity of Ago proteins (Supplementary Figure S3B).

To rigorously validate the first conclusion, we prepared miRNA duplexes whose 3΄ end nt varied (A, U, G or C). These four different miRNA duplexes were programmed in Ago2–RISC and reacted with seedless targets (mm 1–8), which only varied at their 5΄ end nt (Figure 6D). The 3΄ ends of the miRNAs were efficiently destabilized only when their 3΄ end nts were complementary to their corresponding target (*i.e*., A:U, U:A, C:G or G:C) (Figure 6E), as was also confirmed in the other tested miRNA and via endogenous Ago2 in HeLa cell lysates (Supplementary Figure S3C). In addition, we also tested this with miRNAs and their targets that varied in their 3΄ end structures (Figure 6F) or lengths (Supplementary Figure S3D), and the results confirmed that the base-complementarity of the 3΄ end nt is an important determinant of miRNA stability (Figure 6G).

A natural miRNA duplex contains a passenger strand that is often mismatched in the 5΄ seed region of the guide strand, which leads to the question of whether a passenger strand-like target can trigger the 3΄ end destabilization of the guide (Supplementary Figure S4A) Interestingly, the passenger strand-like target did not induce any deleterious effects if the guide strand had at least a 1-nt overhang at its 3΄ end (Supplementary Figure S4B). Consistent with our results regarding the negative impact of the complementarity of the 3΄ end nt, these findings suggest that the 3΄ end of the guide should be freely available to the PAZ domain, not only for optimal RISC assembly (Supplementary Figure S4C), but also for its own stability.

### Dynamic conformational changes of the PAZ domain drive 3΄ end destabilization

How are the 3΄ ends of miRNAs destabilized? Ago proteins experience dynamic conformational changes during RISC assembly and catalysis (38,39). A two-state model was originally proposed by Tomari and Zamore (40), based on their biochemical characterization of Ago proteins, which was later supported by structural studies (41,42). This model postulates that the seed region is first organized into an A-form like arrangement that creates a suitable target binding site. The 3΄ ends of the miRNAs are initially anchored in the PAZ domain, and it dislodges when the miRNA-target duplex propagates toward the 3΄ end of the guide. We hypothesized that 3΄ end destabilization could occur during these structural rearrangements that are associated with target binding. To test this idea, we aimed to determine whether functional disruptions of Ago2 alleviate 3΄ end destabilization.

**Figure 7.**
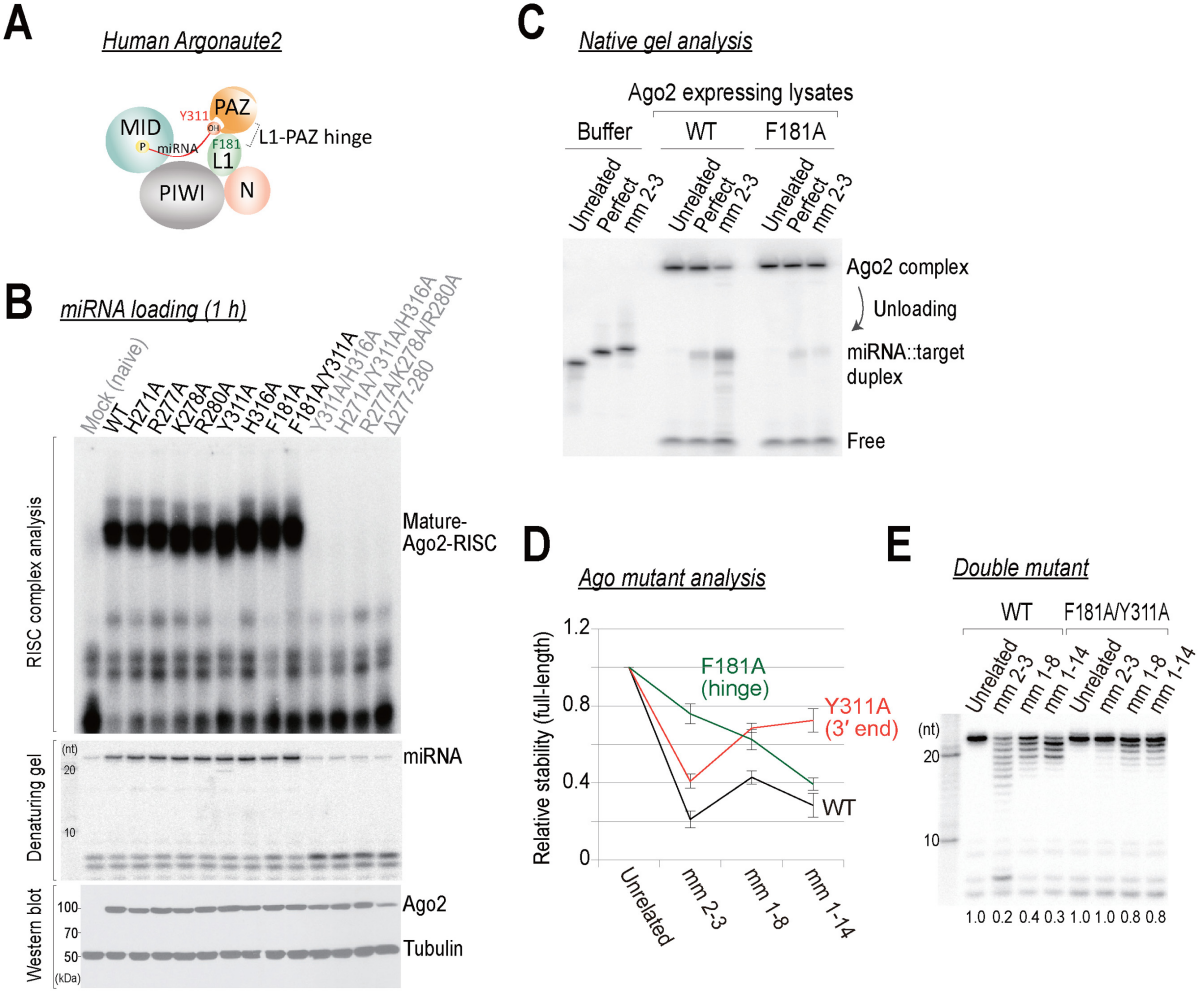
Identification and characterization of human Ago2 domains that are required for 3΄ end destabilization. (**A**) A schematic representation of human Ago2 domains. The miRNA is anchored at its ends in the PAZ and MID domains. (**B**) Top: miRNA duplexes containing radiolabeled guide strands were incubated in lysates expressing tagged wild-type or mutant Ago2 proteins at 37°C for 1 h. The RISC complexes were analyzed in a vertical agarose native gel at 4°C. Middle: miRNAs were analyzed in parallel by 15% denaturing-PAGE. Bottom: western blot analysis using an anti-FLAG antibody confirmed the expression of the tagged Ago2 mutant proteins. Anti-tubulin served as an internal control. (**C**) The F181A hinge mutation drastically inhibits miRNA release from the Ago2-complex. The samples were analyzed by 15% native-PAGE following the *in vitro* destabilization reactions in Ago2-expressing lysates or buffers. (**D** and **E**) The L1 and PAZ domains of human Ago2 are required for 3΄ end destabilization. Data are the mean ± SD for three independent experiments.

First, it has been proposed that the hAgo2 PAZ domain moves like a discrete rigid body along the other domain, and that the ‘hinge’ for this pivotal conformational change resides in the α7–L1 domain (43). Further supporting this line of thought, a recent study showed that hAgo2 PAZ is largely fixed on the tip of L1 and, therefore, it moves in concert with L1 (44). Moreover, we and others previously showed that F181, which resides in the hinge (L1), is required for efficient small interfering RNA (siRNA) duplex separation during RISC assembly (6,45). Therefore, F181 is a candidate for the hinge of the L1-PAZ domain (Figure 7A).

Second, within the PAZ domain, we focused on residues that are expected to interact with the 3΄ end nt of the guide, based on our results (Figure 6E) and structural modeling (Supplementary Figure S5A). To this end, we generated a series of Ago2 mutants (Figure 7B). The Ago2 mutant proteins that were defective in forming miRNA–guide RISC complex (miRISC) were excluded for further analysis (Figure 7B). We found that mutation of the F181 hinge drastically compromised 3΄ end destabilization (Supplementary Figure S5B) and miRNA release from Ago2 (Figure 7C). Additionally, Y311, which is close to the 3΄ end nt of the guide in the PAZ domain, moderately, but reproducibly, reduced the extent of 3΄ end destabilization (Supplementary Figure S5C).

Then, we analyzed these two mutants using several non-canonical targets (Figure 7D and Supplementary Figure S5D). When the seeds were partially matched (mm 2–3), mutation of the hinge largely protected the miRNAs from destabilization, whereas the Y311A mutation mildly increased the level of protection (Figure 7D). In contrast, when the seeds were completely mismatched (mm 1–8 or mm 1–14), the hinge mutant was less able to protect the miRNAs from destabilization, whereas the Y311A mutant strongly inhibited destabilization (Figure 7D). These results indicate that even a partial seed match is helpful for positional shifts in the PAZ domain. In support of this hypothesis, a previous molecular dynamic simulation suggested that a partial mutation in the seed region led to a large bending motion of the PAZ domain along the hinge, which facilitated a target interaction in the 3΄ half of the guide (46). Strikingly, miRNA destabilization was mostly abolished in the F181A/Y311A L1-PAZ double mutant (Figure 7E and Supplementary Figure S5E). Taken together, we concluded that residues in the PAZ domain and related conformational changes are likely to be responsible for target-directed miRNA destabilization.

### Non-canonical target and anti-miR possibly employ distinct mechanisms for miRNA destabilization

Approaches that are based on artificial ASOs have been used to specifically inhibit miRNA function both *in vitro* and *in vivo* (47,48). In addition, a recent study employed 2΄-*O*-methylated anti-miR to recapitulate target-directed miRNA degradation in cultured cells (49). Although the underlying mechanisms are still unclear, it is generally believed that the anti-miR binds with high affinity to the active miRISCs and thereby blocks their binding to endogenous RNA targets, acting as decoy or ‘sponges’. Motivated by these, we compared and evaluated miRNA inhibition potencies of non-canonical target and 2΄-*O*-methylated anti-miR both *in vitro* and in cultured cells.

We first performed *in vitro* miRNA destabilization assay, followed by cleavage assay, as in Figure 5C. The addition of 2΄-*O*-Me anti-miR completely inhibited cleavage activities (47,48) without compromising the stability of miRNAs (Supplementary Figure S6A). This is consistent with previous findings that 2΄-*O*-Me anti-miR can be used to stably capture Ago protein complexes (34,48), indicating that anti-miR do not destabilize miRNAs *in vitro*. In contrast, transfection of 2΄-*O*-Me anti-miR in cultured cells resulted in the dramatic reduction in the level of miRNAs in Ago2 (Supplementary Figure S6B). The discrepancy between two results (*in vitro* and cultured cells) indicates that non-canonical target and anti-miR possibly employ distinct mechanisms for miRNA destabilization (Supplementary Figure S6C).

The results from our *in vitro* system are likely to directly reflect an intrinsic property of Ago proteins; namely, the seedless, non-canonical targets can potentially induce the rearrangements in Ago structure to cause miRNA destabilization, at least in high concentration (>1 μM). Because the non-canonical target has approximately 10-fold less affinity to RISC (Figure 4B), low concentration of the target RNAs in cultured cell-based assay (<0.1 μM) may not be high enough to overcome the reduced binding affinity of Ago2 for targets that contain mismatches in the seed region. The results from cleavage assay indicate that Ago2 may bind and unbind non-canonical targets multiple times (*i.e*., unstable, low affinity binding) (Supplementary Figure S6C). In contrast, anti-miR appears to be stably associated with Ago proteins (*i.e*., stable, high affinity binding) (Supplementary Figure S6C). Intriguingly, the miRNAs were still found in the input fraction upon transfection of anti-miRs in cultured cells (Supplementary Figure S6B). Although the exact mechanism is unclear, we postulate that anti-miR may act either by sequestering the miRNAs without causing significant degradation (50) (possibly at early time points), or by slowly promoting the unloading of miRNAs in Ago proteins, thereby inducing their degradation (51) (Supplementary Figure S6C).

### Non-canonical target-directed mechanism is likely to operate in living cells

**Figure 8.**
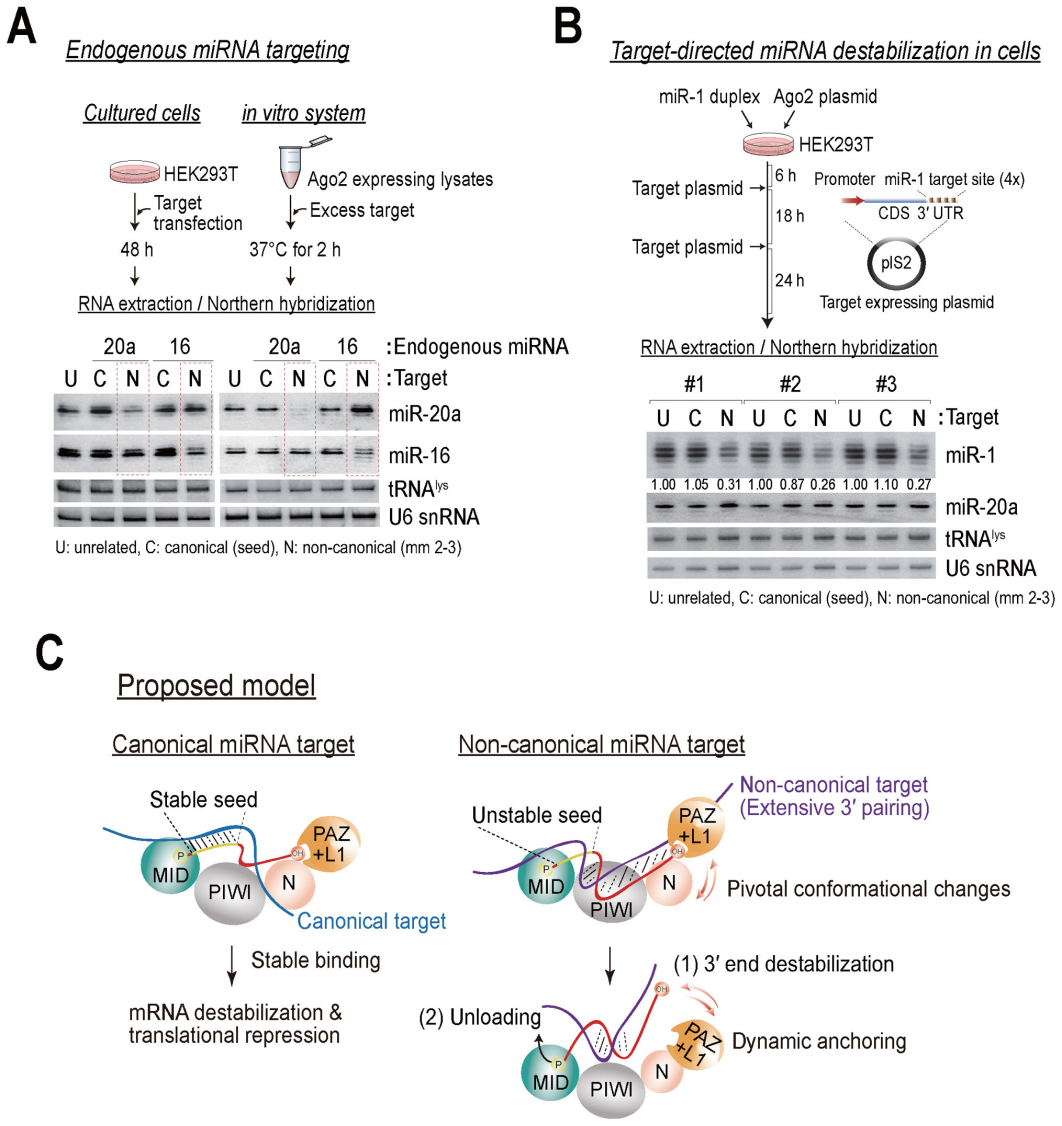
Non-canonical targets destabilize miRNAs in human cells. (**A**) Non-canonical targets specifically decrease the level of the cognate endogenous miRNAs both *in vitro* and in cells. Top: schematic of the experiment. Bottom: endogenous miR-16 and miR-20a were detected by northern blotting and each served as a loading control for the other. Blotting for U6 snRNA and tRNA^lys^ also served as loading controls. U: unrelated, C: canonical (seed), N: non-canonical (mm 2–3). (**B**) miRNAs are destabilized when their non-canonical targets are expressed. Top: schematic of the experiment. Bottom: northern blot analysis of miR-1 after transfection with plasmids encoding unrelated, canonical (seed) or non-canonical (mm 2–3) target sites. The blot was re-probed for miR-20a as a loading control. The numbers below the blot are the relative expression levels, normalized using the miR-20a loading control. Blotting for U6 snRNA and tRNA^lys^ also served as loading controls. Three independent experiments are shown. (**C**) A proposed model for the miRNA destabilization mechanism in human Ago. Left: miRNAs preferentially bind to targets containing intact seed matches, which may allow them to exert their regulatory roles. Right: the 3΄ end destabilization of miRNAs is enhanced by the interaction with non-canonical targets (an unstable seed compensated by extensive 3΄ pairing), and it is driven by the highly dynamic nature of the L1-PAZ domain of human Ago proteins. Unloading may occur during some cases, possibly because of the instability of the miRNA seed region.

There are two sides to non-canonical targets: (i) non-canonical target has a great potential to destabilize miRNAs; and (ii) non-canonical target has reduced binding affinity to RISC because of mismatches in the seed region. This seemingly paradoxical nature of the non-canonical target prompted us to examine what extent our *in vitro* results recapitulate cellular processes. In this regard, we first tested if the addition of non-canonical targets destabilizes endogenous miRNAs that are pre-loaded in Ago proteins both *in vitro* and in cultured cells (Figure 8A). To this end, we targeted the two most abundantly expressed miRNAs (*i.e*., miR-20a and miR-16) in HEK293T cells (52). The HEK293T cells were transfected with 100 nM of the corresponding target RNAs of each miRNA and subjected to northern blot analysis. The results showed that non-canonical targets specifically decrease the level of their cognate miRNAs in living cells (Figure 8A, left), which is largely consistent with the *in vitro* results with saturating target concentrations (1 μM) (Figure 8A, right).

To efficiently recapitulate miRNA destabilization in cells, we prepared 4× miR-1 target plasmids encoding unrelated, canonical (seed) or non-canonical (mm 2–3) target sites. HEK293T cells were initially co-transfected with miR-1 duplex and Ago2 plasmid for efficient exogenous miRNA assembly. It is of note that Ago protein is the primary rate-limiting factor of both *in vitro* and *in vivo* RNAi efficacy (53–56). The target plasmids were then sequentially transfected (Figure 8B). At 48 h post-transfection, RNAs were extracted and subjected to northern blot analysis. The results again showed that the level of miRNAs was significantly and reproducibly reduced by the expression of non-canonical targets, but not by the unrelated or canonical targets (Figure 8B). Although the physiological miRNA target concentration may vary greatly, these collective results strongly indicate that miRNAs can be destabilized by the non-canonical targets both *in vitro* and in cells.

## DISCUSSION

Steady-state regulation of miRNAs and their targets in cells is a dynamic process that should take into account various factors, including the rates of transcription, processing and decay (57). Therefore, without a controlled system, it may be difficult to ascertain the contribution of the different proposed mechanisms for miRNA regulation, as one phenomenon can be masked by the other in cells. In the present study, to pinpoint the target RNA-directed mechanism and to study the molecular underpinning of associated regulatory events, we established an *in vitro* experimental system. One advantage of the *in vitro* system is that it enables a detailed examination of target-directed destabilization at early time points, while data from cell-based experiments are obtained after 24–72 h, at which point the process is often saturated. Based on our results, we propose that miRNA destabilization is enhanced by interactions with non-canonical targets.

Non-canonical binding modes—that is, those with mismatches in the miRNA seed region, but which are often accompanied by auxiliary 3΄ end pairing—were empirically inferred from high-throughput analyses of an miRNA–target chimera, and they were shown to constitute 15–40% of the captured sites (26–30,58). In line with these findings, a molecular simulation study also revealed that mismatches in the seed region are largely allowable without compromising overall Ago-complex stability (46). However, despite their prevalence, non-canonical sites are generally less effective in target silencing than canonical sites (59), which leads to questions regarding their biological roles. Our findings implicate a possible biological role of these non-canonical targets in controlling the stability of miRNAs. We suggest that non-canonical targets are not something to be regulated by miRNAs, but instead, they provide a stability control mechanism in the regulation of miRNAs. We believe that our results may provide a novel insight into the mechanism for stabilizing selection that is expected to maintain a rigid conservation of miRNA seed sequences for the functional and selective targeting.

Currently, no established methods are available to address the genome-wide contribution of a target-directed mechanism (*i.e*., each target to each miRNA). Destabilization effects were mostly diminished when we incubated miRISC with both canonical and non-canonical targets (data not shown). These results suggest that if canonical targets are abundantly expressed in a given cell type, most miRNAs might preferentially bind to those containing intact seed matches (Figure 4B), which may allow them to exert their regulatory roles (Figure 8C). Therefore, a target-directed mechanism may be challenging to generalize, and it should depend on the cell-type specific, spatio-temporally regulated expression of canonical and non-canonical targets. Further studies are needed to determine what extent such mechanisms contribute to the regulation of miRNA function *in vivo*.

An early study hypothesized that miRNAs can be destabilized by targets if their 3΄ ends are released from the PAZ pocket (25). Although further structural studies are needed to extensively validate this hypothesis, we first directly demonstrated *in vitro* that residues in the PAZ domain and its hinge are involved in this process. The 3΄ end of miRNAs that is free from the PAZ domain may then become susceptible to 3΄–5΄ exonuclease or nucleotidyltransferases (49). We found that miRNAs tend to be 3΄ trimmed, rather than tailed, in cytoplasmic lysates from HEK293T or HeLa cells, although the target-directed 3΄ tailing of miRNAs has been observed in several other systems, including neuronal cells (24) and *Drosophila* embryo lysates (25). We believe that the differences are largely attributed to the availability of Ago paralogs and distinctive enzymes (*i.e*., exonucleases or nucleotidyltransferases) within a given system.

Supporting this idea, we were able to observe both 3΄ tailing and trimming of miRNAs in *Drosophila* Ago1 by highly complementary targets in S2 cell lysates (Supplementary Figure S7). However, this was not the case for those bound to *Drosophila* Ago2 (Supplementary Figure S7), because they possesses a 2΄-*O*-methyl group at their 3΄ end, which is catalyzed by endogenous Hen1 (25). Interestingly, however, miRNAs bound to *Drosophila* Ago1 did not appear to interact with seedless 3΄ paired targets (Supplementary Figure S7), which is consistent with a previous study (25). Although the distinct mechanisms remain to be elucidated, we postulate that *Drosophila* Ago1 might depart more rapidly from such targets, compared with human Ago2.

Our findings suggest that a target-directed mechanism can result in two outcomes: unloading and 3΄ end destabilization within Ago proteins. Our results are generally consistent with the analysis by MacRae and colleagues (23), in which unloading is accelerated by mismatches to the 5΄ end and complementarity to the 3΄ end of the guide RNA in recombinant hAgo2 expressed in Sf9 cells. In our experimental system, which consists of the minimal hAgo2–RISC and endogenous proteins present in HEK293T cell lysates, we explored the related molecular mechanisms, and we conclude that such features are essentially linked to the seed region of miRNAs. The only exception was that a fully complementary target did not appear to be effective as previously. Perhaps an additional endogenous protein(s) helps stabilize the interaction between the Ago2-complex and miRNAs containing intact seed matches to the target (60,61).

The unloading mechanism is likely to be the most extreme example of miRNA degradation, because Ago-free ss-miRNAs are susceptible to both 5΄-3΄ or 3΄-5΄ exonuclease(s), and possibly to endonuclease(s). In light of our findings, we propose two consecutive mechanisms of miRNA destabilization (Figure 8C): (i) 3΄ end destabilization is triggered by an extensive 3΄ interaction and the large-scale motion of the L1-PAZ domain, while the MID domain still binds to the 5΄ phosphate end of miRNAs; and (ii) unloading may occur during some cases, possibly because of the instability of the miRNA seed region. The 3΄ end destabilization mechanism may explain the 3΄ heterogeneity of miRNAs. The 3΄ heterogeneity may impact target silencing by influencing the preference for alternative binding sites within target RNAs. Importantly, we showed that 3΄ pairing of a miRNA may influence its own stability, which probably correlates with its silencing efficiency and multiple-turnover (23,62–65). Therefore, the impact and biological consequences of the 3΄ end destabilization mechanism can be diverse and dynamic.

In sum, our findings reflect another layer onto the mutual regulatory circuits between miRNAs and their various targets. Rather than transcriptional control of miRNA biosynthesis, regulation of mature miRNA turnover via unloading and 3΄ end destabilization in Ago proteins serves as a means to refine and diversify miRNAs in cells.

## Supplementary Material

Supplementary Data
