# Supplementary material for: Non-canonical targets destabilize microRNAs in human Argonautes
Source: Nucleic Acids Res. 2017 Jan 23;45(4):1569–83. doi: 10.1093/nar/gkx029 (PMC5389725; doi:10.1093/nar/gkx029)
Supplement: Supplementary Data [file gkx029_Supplementary_Data.zip › nar-03290-y-2016-File003.pdf]

Supplementary Data

Non-canonical Targets Destabilize MicroRNAs in Human Argonautes

June Hyun Park, Sang-Yoon Shin and Chanseok Shin

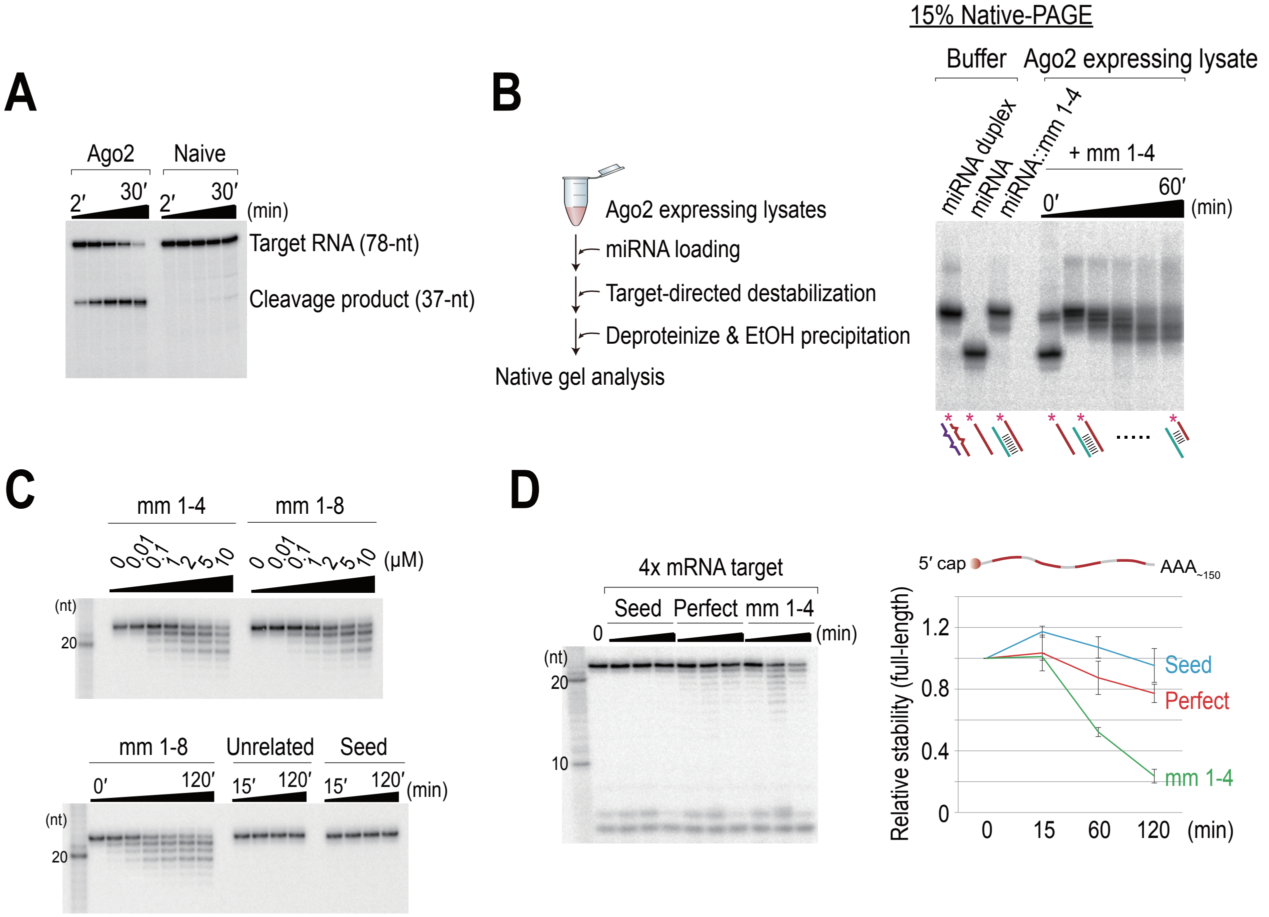

Supplementary Figure S1. ***in vitro*** **recapitulation of target RNA-directed miRNA destabilization in human Argonaute2**

(A) Mammalian cell-free system from HEK293T cells that faithfully recapitulates RNAi *in vitro.* miRNA duplex was assembled in lysates from naive HEK293T cells and from cells expressing hAgo2 for 15 min. Cap-radiolabeled target RNA (78-nt) was then added and further incubated for the indicated times, which yielded a 5′ cleavage product (37-nt), as a diagnostic for hAgo2-mediated catalysis. (B) Left: schematic of the experiment. Right: miRNA-target duplexes are analyzed in 15% native-PAGE following *in vitro* target-directed miRNA destabilization assay. (C and D) Non-canonical targets destabilize miRNAs in Ago2-RISC in a time- and concentration-dependent manner, irrespective of whether the targets are synthetic or *in vitro*-transcribed mRNAs. Data are the mean ± SD for two independent experiments.

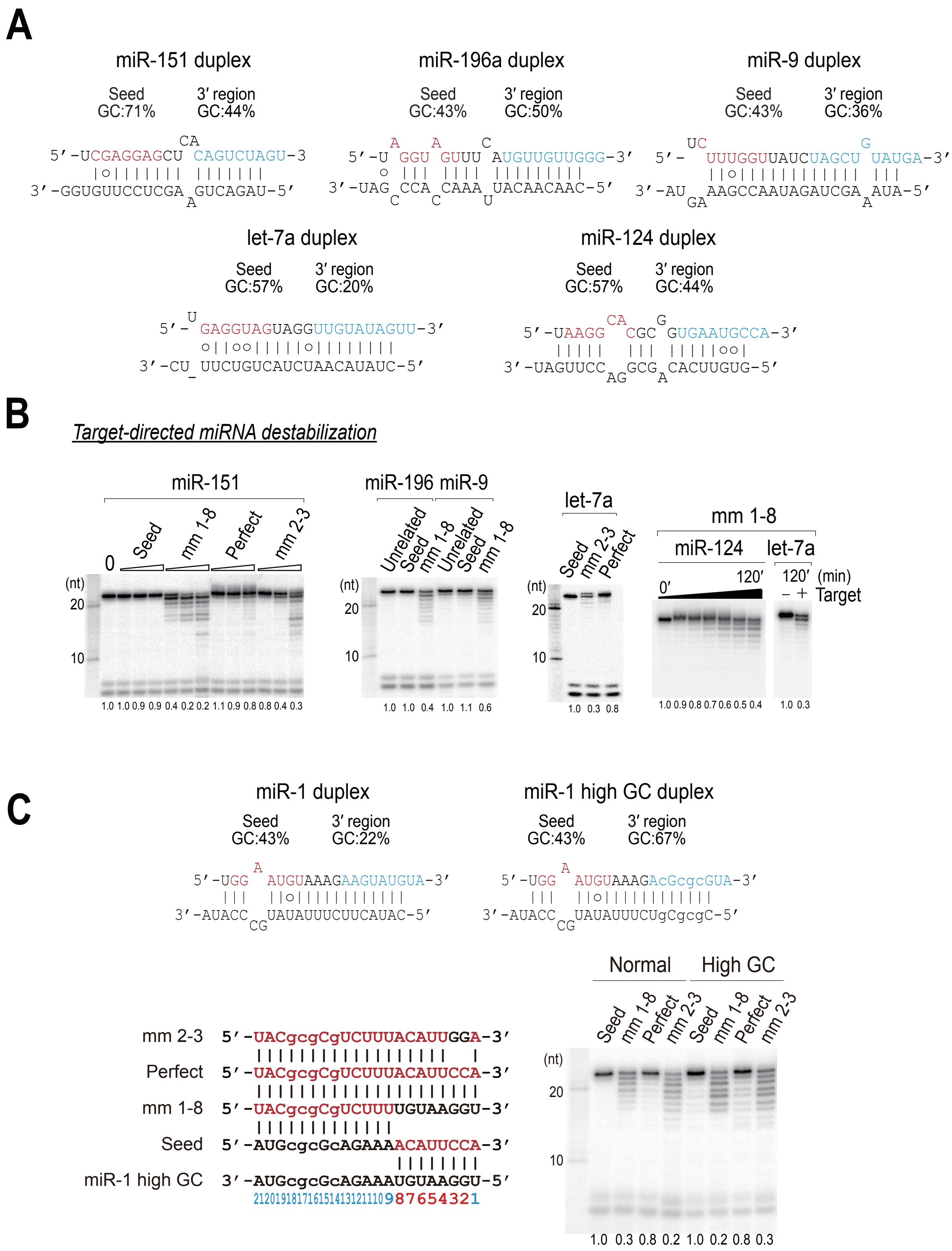

Supplementary Figure S2. Non-canonical targets trigger the 3' end destabilization of many other miRNAs

(A) Various miRNA duplexes used for *in vitro* assay. The GC contents of the seed (g2-8, red) or the 3′ end region (considered as g13-21, blue) are shown. (B) Non-canonical targets destabilize many other miRNAs. (C) The effects of the GC content of the 3′ end region of miRNAs for target-directed destabilization. The 3′ end region of the miR-1 duplex was mutated to have a high GC content (from 22 to 67%).

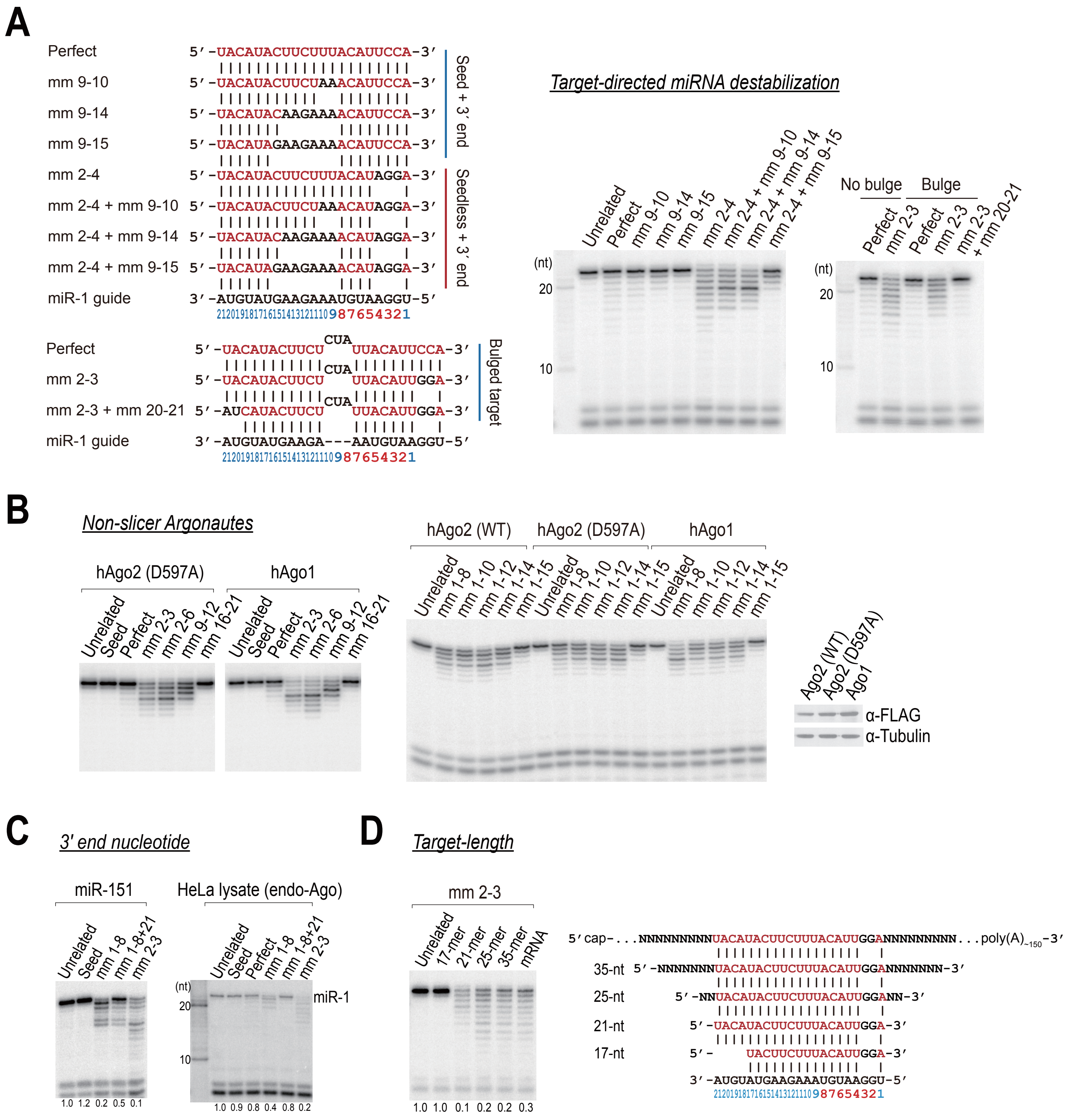

Supplementary Figure S3. 3' complementarity confers specificity for destabilization

(A) Schematic of paired miRNAs and complement target RNAs. Left: 3' end destabilization is mostly diminished when miRNAs are seed matched. Right: 3-nt bulge is tolerable. (B) Human Argonautes are likely to share a similar mechanism for target-directed destabilization, irrespective of their slicer-activity. (C) A mismatch at the 3′ end nucleotide inhibits 3' end destabilization of miR-151 (left) and miR-1 via endogenous Ago2 in HeLa cell lysates (right). (D) 3' complementarity is an important determinant of the 3' end stability, regardless of the lengths of target RNAs.

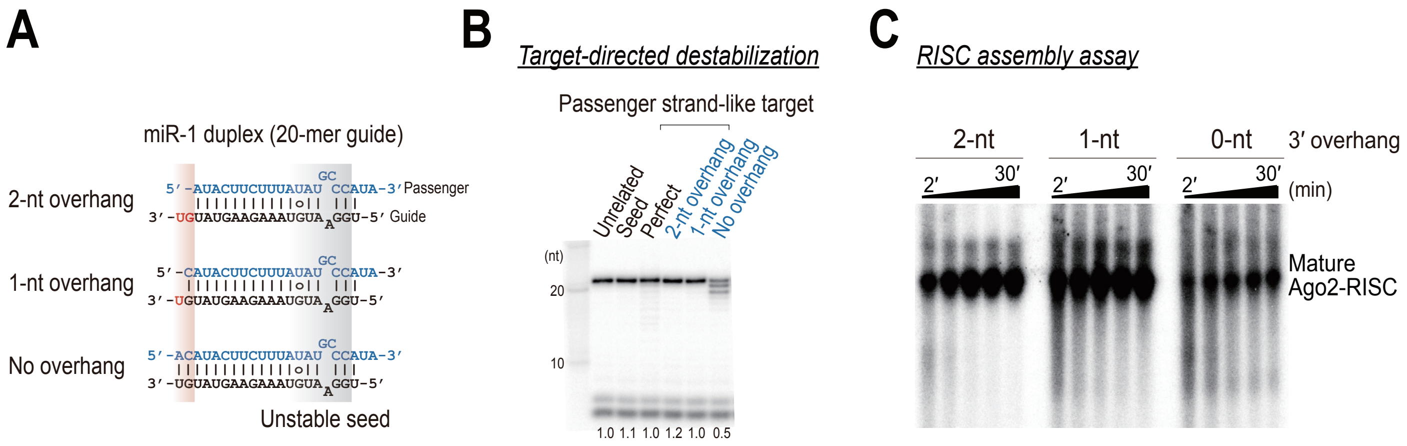

Supplementary Figure S4. Two nucleotide 3′ guide strand overhang is important for miRNA stability and optimal RISC assembly

(A) Schematic of paired miRNAs and passenger strand-like targets. (B) The canonical passenger-strands with 2-nt 3′ overhang do not destabilize miRNAs. (C) miRNA duplexes with 2-nt 3′ overhang are optimal substrate for Ago2-RISC assembly. miRNA duplexes containing radiolabeled guide strands were incubated in lysates expressing tagged Ago2 for the indicated times. The RISC complexes were separated on a vertical agarose native gel at 4°C.

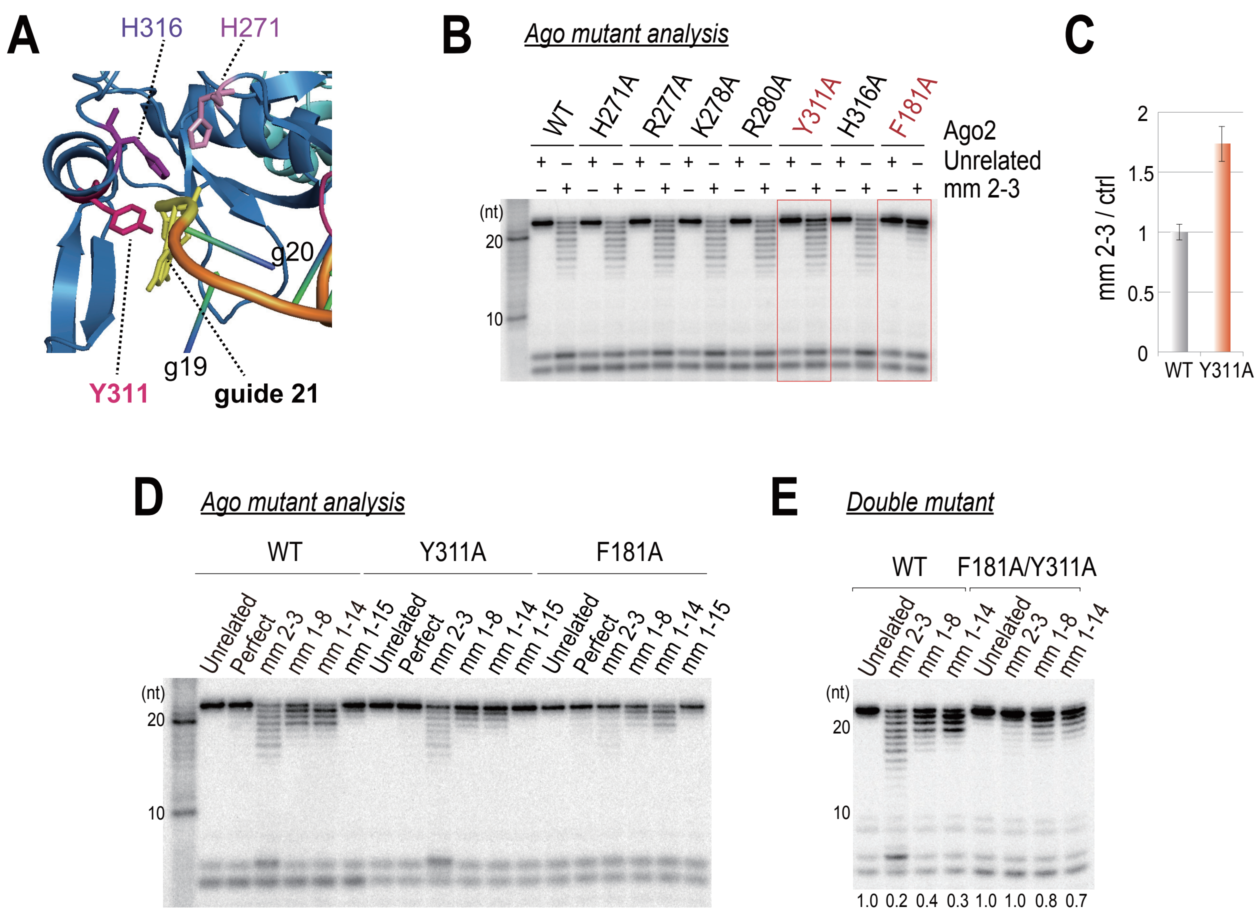

Supplementary Figure S5. Identification and characterization of human Argonaute2 domains that are required for 3' end destabilization

(A) The residues of the hAgo2 PAZ domain that are expected to interact with the 3′ end nt of the guide (snapshot of the structure 4W5N taken in pymol). (B) The residues, Y311 and F181, are involved in the 3′ end destabilization. (C) The Y311 data (mm 2-3/ctrl) are the mean ± SD for three independent experiments. (D) Representative gel data for Figure 7D. (E) Repetitive experiment of Figure 7E.

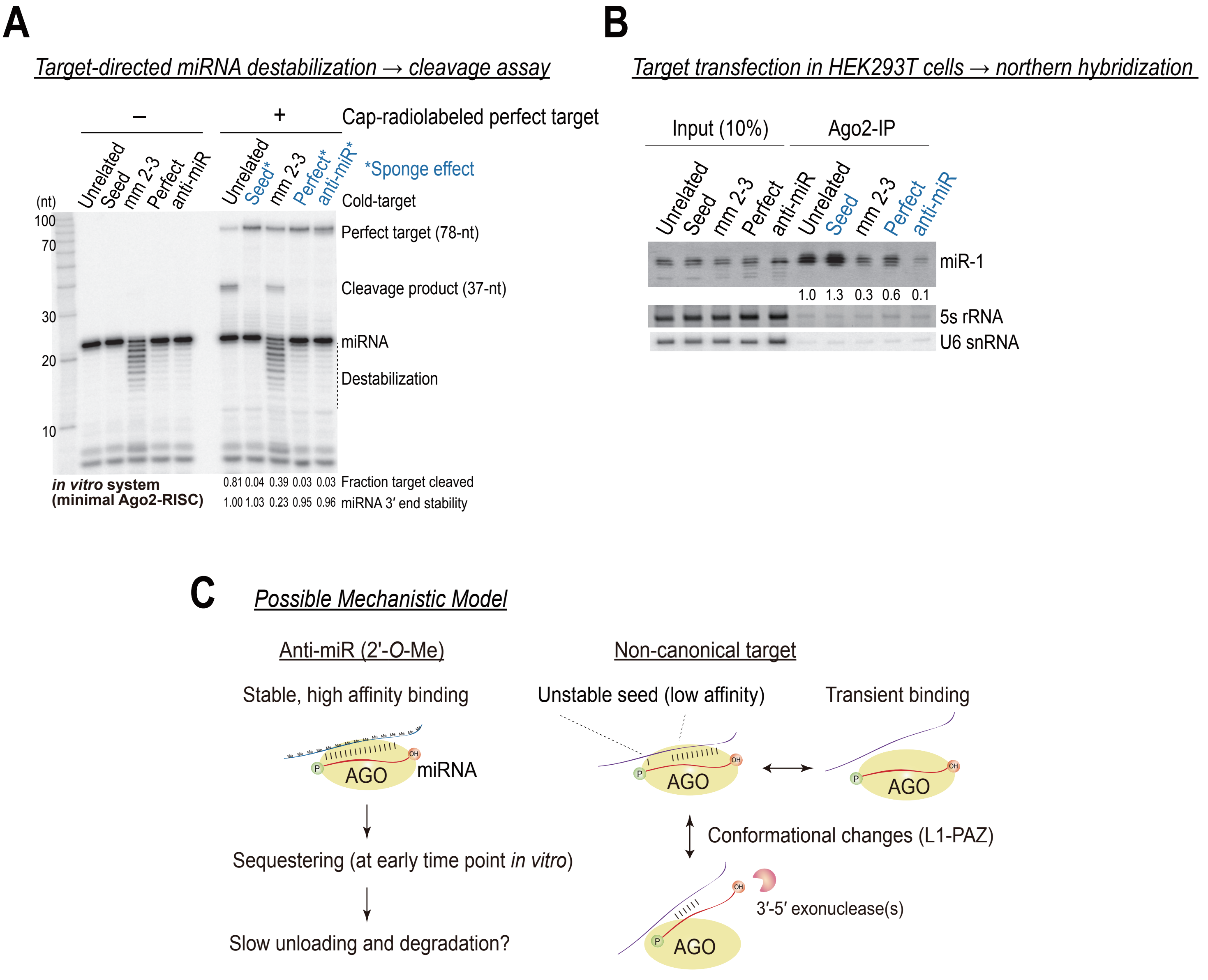

Supplementary Figure S6. Non-canonical target and anti-miR possibly employ distinct mechanisms for miRNA destabilization

(A) The addition of 2'-O-Me anti-miR completely inhibited cleavage activities without compromising the stability of miRNAs. miRNAs were first destabilized by cold targets, prior to the addition of cap-radiolabeled perfect targets (as in Figure 5C). (B) Transfection of 2'-*O*-Me anti-miR in cultured cells resulted in the dramatic reduction in the level of miRNAs in Ago2. HEK293T cells were transfected with 10 nM miRNA duplex, 100 nM target RNA and FLAG-Ago2 expression plasmid. Cell lysates were subjected to Ago2-IP, followed by northern blotting using the miR-1 probe. The blot was probed for U6 snRNA as a loading control. The numbers below the blot are the relative expression levels, normalized using the loading control. Ethidium bromide-stained 5s rRNA served as another loading control. (C) An envisioned model of several distinct action mechanisms of target-directed miRNA destabilization.

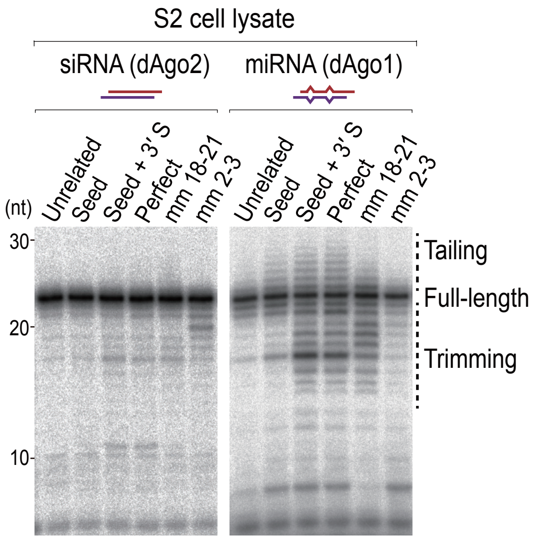

Supplementary Figure S7. Target-directed tailing and trimming of miRNAs in *Drosophila* S2 cell lysates

The *in vitro* target-directed destabilization assay (as in Figure 2A) was performed in *Drosophila* S2 cell lysates at 25°C. The different small RNA duplexes were used to favor the assembly of miRNA duplexes into dAgo1-RISC or siRNA duplexes into dAgo2-RISC [Tomari, Y., Du, T. and Zamore, P.D. (2007) Sorting of Drosophila small silencing RNAs. *Cell*, 130, 299-308.].

Supplementary Table 1. The sequences of oligonucleotides (5′-3′)

Site-directed mutagenesis

H271A Fwd: GCCTGTGGGCAGATGAAGAGGAAGT
H271A Rev: CGTTATCTCCACCTTTAGACCTTTAAT
R277A Fwd: CTTCGCCTTCATCTGCCCACAGTGCGTTATC
R277A Rev: TACCGCGTCTGCAATGTGACCCGGCGGC
K278A Fwd: CGCCCTCTTCATCTGCCCACAGTGCGTTATC
K278A Rev: TACCGCGTCTGCAATGTGACCCGGCGGC
R280A Fwd: CTTCCTCTTCATCTGCCCACAGTGCGTTATC
R280A Rev: TACGCCGTCTGCAATGTGACCCGGCGGC
Y311A Fwd: GACAGGCACAAGTTGGTTCTGCGCTACC
Y311A Rev: CTTGAAAGCCTGGGCCACCGTGCACTC
H316A Fwd: GACAGGGCCAAGTTGGTTCTGCGCTACC
H316A Rev: CTTGAAATACTGGGCCACCGTGCACTC

Northern hybridization DNA probe

miR-1: TACATACTTCTTTACATTCCA
miR-16: CGCCAATATTTACGTGCTGCTA
miR-20a: CTACCTGCACTATAAGCACTTTA
miR-151: ACTAGACTGTGAGCTCCTCGA
let-7a: AACTATACAACCTACTACCTCA
$tRNA^{lys}$: GAGATTAAGAGTCTCATGCTC
U6 snRNA: TTGCGTGTCATCCTTGCGCAGG

MicroRNA

miR-1 guide: UGGAAUGUAAAGAAGUAUGUA
miR-1 guide 3'U: UGGAAUGUAAAGAAGUAUGUU
miR-1 guide 3'G: UGGAAUGUAAAGAAGUAUGUG
miR-1 guide 3'C: UGGAAUGUAAAGAAGUAUGUC
miR-1 guide 20 nt: UGGAAUGUAAAGAAGUAUGU
miR-1 guide 22 nt: UGGAAUGUAAAGAAGUAUGUAU
miR-1 high GC guide: UGGAAUGUAAAGACGCGCGUA
miR-1 passenger: CAUACUUCUUUAUAUGCCCAUA
miR-1 passenger 21 nt: AUACUUCUUUAUAUGCCCAUA
miR-1 passenger 23 nt: ACAUACUUCUUUAUAUGCCCAUA
miR-1 high GC passenger: CGCGCGUCUUUAUAUGCCCAUA
miR-151 guide: UCGAGGAGCUCACAGUCUAGU
miR-151 passenger: UAGACUGAAGCUCCUUGUGG
miR-196 guide: UAGGUAGUUUCAUGUUGUUGGG
miR-196 passenger: CAACAACAUUAAACCACCCGAU
miR-9 guide: UCUUUGGUUAUCUAGCUGUAUGA
miR-9 passenger: AUAAAGCUAGAUAACCGAAAGUA
let-7a guide: UGAGGUAGUAGGUUGUAUAGUU
let-7a passenger: CUAUACAAUCUACUGUCUUUC
miR-124 guide: UAAGGCACGCGGUGAAUGCCA
miR-124 passenger: GUGUUCACAGCGGACCUUGAU

Target RNA

Unrelated: GCAUUCACCGCGUGCCUUAAU
miR-1 seed: AUGUAUGAAGAAAACAUUCCA
miR-1 seed + 3′s: AUGUAACUUGAAAACAUUCCA
miR-1 perfect: UACAUACUUCUUUACAUUCCA
miR-1 mm 1-8: UACAUACUUCUUUUGUAAGGU
miR-1 mm 1-4: UACAUACUUCUUUACAUAGGU
miR-1 mm 5-8: UACAUACUUCUUUUGUAUCCA
miR-1 mm 9-12: UACAUACUUGAAAACAUUCCA
miR-1 unrelated(mm 2-4): AUGUAUGAAGAAAACAUAGGA
miR-1 mm 2-3: UACAUACUUCUUUACAUUGGA
miR-1 mm 3-4: UACAUACUUCUUUACAUAGCA
miR-1 mm 4-5: UACAUACUUCUUUACAAACCA
miR-1 mm 5-6: UACAUACUUCUUUACUAUCCA
miR-1 mm 6-7: UACAUACUUCUUUAGUUUCCA
miR-1 mm 7-8: UACAUACUUCUUUUGAUUCCA
miR-1 mm 2-4: UACAUACUUCUUUACAUAGGA
miR-1 mm 2-5: UACAUACUUCUUUACAAAGGA
miR-1 mm 2-6: UACAUACUUCUUUACUAAGGA
miR-1 mm 18-21: AUGUUACUUCUUUACAUUCCA
miR-1 mm 16-21: AUGUAUCUUCUUUACAUUCCA
miR-1 mm 9-12 + mm 21: AACAUACUUGAAAACAUUCCA
miR-1 mm 9-12 + mm 20-21: AUCAUACUUGAAAACAUUCCA
miR-1 mm 9-12 + mm 19-21: AUGAUACUUGAAAACAUUCCA
miR-1 mm 9-12 + mm 17-21: AUGUAACUUGAAAACAUUCCA
miR-1 mm 1-8 + mm 21: AACAUACUUCUUUUGUAAGGU
miR-1 mm 1-8 + mm 20-21: AUCAUACUUCUUUUGUAAGGU
miR-1 mm 1-8 + mm 19-21: AUGAUACUUCUUUUGUAAGGU
miR-1 mm 1-8 + mm 17-21: AUGUAACUUCUUUUGUAAGGU
miR-1 mm 9-10: UACAUACUUCUAAACAUUCCA
miR-1 mm 9-14: UACAUACAAGAAAACAUUCCA
miR-1 mm 9-15: UACAUAGAAGAAAACAUUCCA
miR-1 mm 9-16: UACAUUGAAGAAAACAUUCCA
miR-1 mm 2-4 + mm 9-10: UACAUACUUCUAAACAUAGGA
miR-1 mm 2-4 + mm 9-14: UACAUACAAGAAAACAUAGGA
miR-1 mm 2-4 + mm 9-15: UACAUAGAAGAAAACAUAGGA
miR-1 perfect bulge: UACAUACUUCUCUAUUACAUUCCA
miR-1 mm 2-3 bulge: UACAUACUUCUCUAUUACAUUGGA
miR-1 mm 2-3 + mm 20-21: AUCAUACUUCUCUAUUACAUUGGA
miR-1 mm 2-3 17 nt: UACUUCUUUACAUUGGA
miR-1 mm 2-3 25 nt: UAUACAUACUUCUUUACAUUGGAUA
miR-1 mm 1-10: UACAUACUUCUAAUGUAAGGU
miR-1 mm 1-12: UACAUACUUGAAAUGUAAGGU
miR-1 mm 1-14: UACAUACAAGAAAUGUAAGGU
miR-1 mm 1-15: UACAUAGAAGAAAUGUAAGGU
miR-1 mm 1-16: UACAUUGAAGAAAUGUAAGGU
miR-1 mm 1-8 5'A: AACAUACUUCUUUUGUAAGGU
miR-1 mm 1-8 5'C: CACAUACUUCUUUUGUAAGGU
miR-1 mm 1-8 5'G: GACAUACUUCUUUUGUAAGGU
miR-1 mm 1-8 3' end structure-1: ACAUACUUCUUUUGUAAGGU
miR-1 mm 1-8 3' end structure-2: UACAUACUUCUUUUGUAAGGU
miR-1 mm 1-8 3' end structure-3: AUACAUACUUCUUUUGUAAGGU

miR-1 high GC seed: AUGCGCGCAGAAAUGUAAGGU
miR-1 high GC mm 1-8: UACGCGCGUCUUUUGUAAGGU
miR-1 high GC perfect: UACGCGCGUCUUUACAUUCCA
miR-1 high GC mm 2-3: UACGCGCGUCUUUACAUUGGA
miR-151 seed: UGAUCUGACACUCCUCCUCGA
miR-151 perfect: ACUAGACUGUGAGCUCCUCGA
miR-151 mm 2-3: ACUAGACUGUGAGCUCCUGCA
miR-151 mm 1-8: ACUAGACUGUGAGGAGGAGCU
miR-151 mm 1-8 + mm 21: UCUAGACUGUGAGGAGGAGCU
miR-196 seed: GGGUUGUUGUACUUACUACCUA
miR-196 mm 1-8: CCCAACAACAUGAAUGAUGGAU
miR-9 seed: AGUAUGUCGAUCUAUACCAAAGA
miR-9 mm 1-8: UCAUACAGCUAGAUAUGGUUUCU
let-7a seed: UGAUAUGUUGGAUCUACCUCA
let-7a mm 2-3: ACUAUACAACCUACUACCAGA
let-7a mm 1-8: ACUAUACAACCUAGAUGGAGU
let-7a perfect: ACUAUACAACCUACUACCUCA
miR-124 mm 1-8: UGGCAUUCACCGCCACGGAAU
Unrelated 35 nt: UACACAUGCAUUCACCGCGUGCCUUAAUAUCAGAU
miR-1 seed 35 nt: UACACAUAUGUAUGAAGAAAACAUUCCAAUCAGAU
miR-1 mm 2-3 35 nt: UACACAUUACAUACUUCUUUACAUUGGAAUCAGAU
miR-1 perfect 35 nt: UACACAUUACAUACUUCUUUACAUUCCAAUCAGAU
anti-miR-1: $U_mA_mC_mA_mC_mA_mU_mU_mA_mC_mA_mU_mA_mC_mU_mU_mC_mU_mU_mU_mA_mC_mA_mU_mU_mC_mC_mA_mA_mU_mC_mA_mG_mA_mU_m$
miR-16 seed: GCGGUUAUAAAUGCUGCUGCUA
miR-16 mm 2-3: CGCCAAUAUUUACGUGCUGGAA
miR-20a seed: GAUGGACGUGAUAUUGCACUUUA
miR-20a mm 2-3: CUACCUGCACUAUAAGCACUAAA

ASO for RISC assembly

Unrelated: $U_mC_mU_mU_mC_mG_mC_mA_mU_mU_mC_mA_mC_mC_mG_mC_mG_mU_mG_mC_mC_mU_mU_mA_mA_mU_mA_mC_mC_mU_mU_m$
Seed: $U_mC_mU_mU_mC_mA_mU_mG_mU_mA_mU_mG_mA_mA_mG_mA_mA_mA_mA_mC_mA_mU_mU_mC_mC_mA_mA_mC_mC_mU_mU_m$
mm 2-3: $U_mC_mU_mU_mC_mU_mA_mC_mA_mU_mA_mC_mU_mU_mC_mU_mU_mU_mA_mC_mA_mU_mU_mG_mG_mA_mA_mC_mC_mU_mU_m$
Perfect: $U_mC_mU_mU_mC_mU_mA_mC_mA_mU_mA_mC_mU_mU_mC_mU_mU_mU_mA_mC_mA_mU_mU_mC_mC_mA_mA_mC_mC_mU_mU_m$

4x target mRNA sequences for *in vitro* transcription and cloning

miR-1 unrelated: CGAGAGCTCGCATTCACCGCGTGCCTTAATGTCGAGGCATTCACCGCGTGCCTTAATGTC-GAGGCATTCACCGCGTGCCTTAATGTCGAGGCATTCACCGCGTGCCTTAATACTAGTCGA
miR-1 seed: CGAGAGCTCATGTATGAAGAAAACATTCCAGTCGAGATGTATGAAGAAAACATTCCAGTC-GAGATGTATGAAGAAAACATTCCAGTCGAGATGTATGAAGAAAACATTCCAACTAGTCGA
miR-1 perfect: CGAGAGCTCTACATACTTCTTTACATTCCAGTCGAGTACATACTTCTTTACATTCCAGTC-GAGTACATACTTCTTTACATTCCAGTCGAGTACATACTTCTTTACATTCCAACTAGTCGA
miR-1 mm 1-4: CGAGAGCTCTACATACTTCTTTACATAGGTGTCGAGTACATACTTCTTTACATAGGTGTC-GAGTACATACTTCTTTACATAGGTGTCGAGTACATACTTCTTTACATAGGTACTAGTCGA
miR-1 mm 2-3: CGAGAGCTCTACATACTTCTTTACATTGGAGTCGAGTACATACTTCTTTACATTGGAGTC-GAGTACATACTTCTTTACATTGGAGTCGAGTACATACTTCTTTACATTGGAACTAGTCGA
